# Ovulatory-Based FET Cycles May Achieve Higher Pregnancy Rates in the General Population and among Anovulatory Women

**DOI:** 10.3390/jcm10040703

**Published:** 2021-02-11

**Authors:** Nardin Aslih, Dore Dorzia, Yuval Atzmon, Daniella Estrada, Adrian Ellenbogen, Asaf Bilgory, Einat Shalom-Paz

**Affiliations:** IVF Unit, Hillel Yaffe Medical Center, Hadera 3810101, Israel; nardin_aslih@yahoo.com (N.A.); doreblue101@aol.com (D.D.); atzmony@gmail.com (Y.A.); deg03dana@hotmail.com (D.E.); ellenbogen55@gmail.com (A.E.); asaf_bil@hotmail.com (A.B.)

**Keywords:** PCOS, aromatase inhibitor, frozen embryo transfer, endometrial preparation, natural cycle embryo transfer, artificial cycle embryo transfer

## Abstract

This study evaluated which endometrial preparation protocol in frozen embryo transfer (FET) cycles provides the best results for polycystic ovarian syndrome (PCOS) patients and the general population. This retrospective study of 634 FET cycles was conducted 2016–2018. Cycles were divided into Group A: Artificial endometrial preparations for FET (aFET; *n* = 348), Group B: Ovulatory cycle (*n* = 286) to compare two methods of endometrial preparation for FET. Artificial endometrial preparation with exogenous estrogen and progesterone versus natural ovulation cycles, modified natural cycles using hCG for the final triggering and letrozole-induced ovulation with hCG. Anovulatory patients were analyzed separately. Anovulatory PCOS patients had significantly higher pregnancy rates with letrozole treatment compared with aFET cycles (44% vs. 22.5%; *p* = 0.044). For the entire cohort, ovulatory cycles and aFET were similar in terms of patient characteristics, demographics, infertility causes, treatment protocols and number of embryos transferred. Although the mean ESHRE score of the transferred embryos was higher in the aFET group, we found higher clinical pregnancy rate in the ovulatory cycle FET (41.3% vs. 27.3%, *p* < 0.0001). A better pregnancy rate was found after ovulatory cycle FET. In the ovulatory cycles, the outcome of letrozole-induced and non-induced cycles were comparable. PCOS patients, as well as the general population, may benefit from ovulation induced FET cycles, with significantly better outcomes in FET in ovulatory cycles.

## 1. Introduction

The worldwide shift towards frozen embryo transfer (FET) has accelerated, as more frozen embryos are currently available per patient for future use [1]. Multiple factors contributed to this change, beginning primarily with improved incubators and changes in the mode of embryo preservation towards vitrification, which improved the survival rate and quality of the thawed embryos [2]. The indications for embryo freezing for future use were extended beyond patients with suspected ovarian hyperstimulation syndrome. Cases in which progesterone was elevated during the late follicular phase, abnormal endometrial development and the need for pre-gestational testing are common causes that contributed to the increase in FET cycles and enable optimal Assisted Reproductive Technologies (ART) results in these cases [3].

It is well-established that the window of implantation is a narrow period during the menstrual cycle and that endometrial receptivity must match the embryo’s developmental stage. It is mostly important to schedule the transfer to the most accurate timing of endometrial preparation after progesterone exposure [4]. FET cycles have comparable results to fresh cycles [5,6]; however, there is no consensus regarding the optimal preparation protocol. Approaches to FET cycles include artificial cycles using a combination of estrogen and progesterone in different administration routes, or ovulatory-based treatments that aim to achieve optimal endometrial preparation for embryo transfer [7,8,9,10].

Letrozole became a common drug treatment for inducing ovulation in anovulatory women for insemination cycles, with very good results [11]. Its use was well-established for breast cancer patients undergoing In Vitro Fertilization (IVF) cycles for fertility preservation and results were comparable to those of control patients [12]. A few studies evaluated the outcome of letrozole use in FET cycles, with conflicting results between letrozole and other approaches to endometrial preparation [13,14,15]. However, no consensus was achieved regarding the best protocol for FET [7].

One of the most challenging populations to treat with FET cycles is patients who have polycystic ovarian syndrome (PCOS). PCOS is the most common endocrine cause of anovulatory infertility. In May 2003, the Rotterdam Criteria defined it as exhibiting two of the following three features (after excluding related disorders): (1) oligo- or anovulation, (2) clinical and/or biochemical signs of hyperandrogenism, or (3) polycystic ovaries [16]. In essence, this group of patients usually treated with artificial FET (aFET) because they rarely have ovulatory cycles.

The aims of this study were to compare aFET and ovulatory-based FET among the general population and PCOS patients, and to investigate the impact of letrozole on FET cycle outcomes, including fetal and maternal complications by following the patients until delivery.

## 2. Materials and Methods

### 2.1. Patients

This retrospective cohort study was conducted in a single reproductive center. Records of all patients and their cryopreserved embryos from cycles conducted from 2016 through 2018 were analyzed. Data collection included baseline parameters of age, body mass index (BMI) (kg/m^2^), type of infertility, parity, lifestyle and cause of infertility, as well as treatment parameters, including number of embryos transferred, hormonal level before transfer, embryo morphokinetic scoring by time lapse, endometrial thickness (mm) before transfer and pregnancy outcomes.

To reflect the broad range of patients typically encountered in clinical practice, no inclusion/exclusion criteria were applied regarding baseline characteristics. Only cycles in which transfers were cancelled due to endometrial polyps, premature progesterone elevation and the use of donor oocytes were not included. Anovulatory PCOS patients were analyzed both separately and as part of the cohort.

### 2.2. Embryo Quality Assessment

All oocytes aspirated in the preceding fresh IVF treatment were fertilized and cultured on EmbryoSlides and incubated in the EmbryoScope™ (Unisense FertiliTech, Aarhus, Denmark) up to 5 days with 5.8% CO_2_ at 37 °C and 5% O_2_. Images were acquired of each embryo every 10 min in seven focal planes, starting from the second polar body extraction up to 120 h after fertilization, to determine the exact timing of cell divisions. On day 3, all embryos were scored according to Known Implantation Data (KID) and Alfa ESHRE scores [17]. Only top-quality embryos were vitrified and used in the FET cycles evaluated in this study. Top-quality embryos included KID scores of 5;3 or 5;2 and Alfa ESHRE scores of 4;3 or 4;2.

### 2.3. Treatment Protocol

All patients scheduled for FET arrived during early follicular phase for their first ultrasound evaluation. At that point, it was the physician’s preference to allocate the patients either for artificial frozen embryo transfer (aFET) or for natural cycles (NC-FET). The decision regarding the protocol and medication used was based on the patient’s records, previous cycles, medical history and patient’s preferences, if possible.

### 2.4. Artificial FET Protocol (aFET)

Estradiol 2 mg TID (Estrofem^®^ Novo Nordisk, Bagsværd, Denmark) was started on day 3 of menstruation for at least 8 days. On day 8, the second visit, the endometrium was assessed by transvaginal ultrasound (TVS). When endometrial thickness was more than 8 mm, progesterone was started. The options for progesterone were oral dydrogesterone 10 mg TID (Duphastone^®^ Abbott, Chicago, IL, United States), vaginal micronized progesterone (MVP) 100 mg TID (Endometrin^®^ Ferring, Caesarea, Israel) or MVP gel 90 mg BID (Crinone^®^ 8%, Merck-Serono, Darmstadt, Germany), daily.

The duration of progesterone administration before embryo transfer was according to the embryo’s age. Cleavage stage embryos were transferred after 4 days of progesterone and blastocysts were transferred after 6 days of progesterone administration [10].

Luteal phase support used the above combination of estradiol 2 mg TID and progesterone, as started for endometrial preparation.

### 2.5. Ovulatory Cycle protocols (Ovu-FET)

In ovulation-based cycles, ovulation was either natural or letrozole-induced. Further on, ovulation was natural or triggered by 250 mcg of recombinant hCG (Ovitrelle^®^; Merck-Serono, Darmstadt, Germany) when the leading follicle was 18 mm and endometrial thickness was more than 7.5 mm, as seen on TVS.

In letrozole-induced cycles, patients were treated with 2.5 mg letrozole (Letrozole^®^ Teva, Netanya, Israel) BID for 5 days, from day 5 to day 9 of menstruation. They were followed with TVS until a dominant follicle reached 18 mm and endometrial thickness was more than 7.5 mm.

In all cases, the decision of either a fully natural cycle with spontaneous ovulation or ovulation triggering with Ovitrelle^®^ was based on the timing of the transfer (avoiding weekend transfers) and based on blood levels of luteinizing hormone and appropriate follicular diameter.

The luteal phase support included either oral dydrogesterone 10 mg TID (Duphastone^®^ Abbott, Chicago, IL, United States), vaginal micronized progesterone (MVP) 100 mg TID (Endometrin^®^, Ferring, Caesarea, Israel), or MVP gel 90 mg BID (Crinone^®^ 8%, Merck Serono, Darmstadt, Germany), daily.

### 2.6. Pregnancy Determination

Chemical pregnancy was determined when β-hCG was >50 mIU/mL 14 days after embryo transfer. A clinical pregnancy was confirmed when a gestational sac with fetal heartbeat activity was visible on ultrasound examination at 6 weeks of gestation. Demographic data, treatment information and results, and pregnancy follow-up and outcomes were recorded and monitored until delivery.

### 2.7. Statistical Analysis

We analyzed the entire FET cohort, comparing ovulatory and artificial cycles. We further conducted two sub-analyses: aFET only compared to letrozole-induced Ovu-FET cycles and letrozole-induced Ovu-FET compared to non-induced Ovu-FET cycles.

Statistical analysis was performed using the SPSS software package (SPSS Inc., Chicago, IL, USA). We used the Shapiro–Wilk test to evaluate the distribution of the data. Comparisons were analyzed using Student’s *t* test or a Mann–Whitney U test, each when appropriate. Proportions were compared using a Chi-Square test or Fisher exact test. *p*-values less than 0.05 were considered significant.

A multivariate stepwise regression analysis was conducted to evaluate the impact of the treatment protocol for FET, maternal age, BMI, endometrial thickness, and number of transferred embryos on conception.

The null hypothesis (h0) was that there was no difference in pregnancy rates between aFET and Ovu-FET. The alternative (h1) was that there would be a difference between the two groups of at least 10%. As this was a retrospective evaluation, we included all treatment cycles eligible for the study. We calculated power analysis (post hoc) with www.openepi.com. We wanted to detect the differences in pregnancy and live birth rates between the aFET and Ovu-FET groups at a significance level (alpha) of 0.05, 95% confidence interval. We found that this study had a power of 94% to detect a difference between the groups.

## 3. Results

The total cohort included 634 FET cycles that were reviewed and divided into ovulatory based cycles and aFET. Demographic data were similar. Except for more anovulatory patients in the aFET group, other causes of infertility, fresh cycle characteristics, and number of embryos transferred revealed no significant differences between patients (Table 1).

### 3.1. Ovu-FET Cycles vs. aFET

Despite significantly better morphokinetic scores of embryos transferred in the aFET group (ESHRE score 2.37 ± 0.81 vs. 2.17 ± 0.71, *p* = 0.031), higher chemical, clinical pregnancy rates, and ongoing pregnancy and delivery rates were achieved in the ovulatory cycle-FET group (45.8% vs. 34.2%, *p* = 0.03; 41.3% vs. 27.3%, *p* < 0.0001, and 33% vs. 19%, *p* < 0.0001, respectively; Table 1) and a significantly higher miscarriage rate were observed in the aFET group (54.7% vs. 33%, *p* < 0.0001). Thus, our alternative hypothesis was confirmed.

Analyzing pregnancy outcomes revealed similar numbers of ectopic pregnancies and pregnancy complications, as well as no difference in terms of maternal and fetal morbidity (Table 1).

### 3.2. Letrozole vs. aFET

In a sub-analysis of aFET cycles and letrozole-induced Ovu-FET, the significant difference in clinical pregnancy rates remained (41.3% vs. 27.3%, respectively, *p* = 0.021; Table 2). Pregnancy outcomes resulted in significantly favorable ongoing pregnancy and delivery rates in the letrozole as compared to the aFET group (30% vs. 19%, *p* = 0.04; Table 2).

### 3.3. Letrozole-Induced Ovulation vs. Noninduced NC-FET

We divided the Ovu-FET cycles into two sub-groups based on the administration of medication for ovulation induction (letrozole-induced or non-induced natural ovulation) (Table 3). Endometrial thickness was significantly lower in the letrozole-induced group (8.1 ± 1.9 mm vs. 9.2 ± 2.2 mm, *p* = 0.003). However, no difference was found in rates of pregnancy, miscarriage, or pregnancy complications. Only fetal birth weights were significantly higher in non-induced Ovu-FET as compared to letrozole-induced NC-FET (3266 g vs. 2617 g, *p* = 0.008; Table 3).

### 3.4. Anovulatory PCOS Patients

A total of 105 FET cycles in PCOS patients were evaluated. They were divided into two treatment protocols: 80 with FET and 25 with letrozole. Patient parameters were comparable between groups; however, the clinical pregnancy rate was significantly higher in the letrozole-induced Ovu-FET compared to aFET (44% vs. 22.5%, *p* = 0.044; Table 4).

###  3.5. Multivariate Regression Analysis

Multivariate regression analysis revealed that the treatment protocol used for endometrial preparation, maternal age, and number of transferred embryos were the only parameters that predicted likelihood of conception. The maternal age, protocol used, and the number of embryos transferred had significant effects on pregnancy rate. The treatment protocol based on Ovu-FET for endometrial preparation achieved significantly better outcomes, as compared to artificial cycles (OR = 1.896, 95%CI = 1.31–2.73, *p* = 0.001). Letrozole treatment achieved significantly better results overall and for PCOS patients, as compared to artificial cycles (OR = 2.029, 95%CI = 1.210–3.402, *p* = 0.007). For each additional year of maternal age, OR = 0.967, 95%CI = 0.942–0.993, *p* = 0.015, and the OR for each additional embryo transferred was 1.518, 95%CI = 1.004–2.297, *p* = 0.048. BMI, endometrial thickness, and embryo quality did not affect pregnancy rate.

## 4. Discussion

This study aimed to determine which FET protocol would achieve the best pregnancy outcomes. We found that for PCOS patients, letrozole-induced treatment outcomes had significantly better pregnancy rates. Moreover, for the general population, induced-treatment cycles based on ovulation resulted in significantly higher pregnancy rates, regardless of whether ovulation was natural or induced with letrozole, as compared with artificial induced cycles. The clinical pregnancy rates were comparable between letrozole-induced ovulation and natural ovulatory cycles.

The number of FET cycles has increased dramatically over time due to great improvements in freezing technologies and the vitrification process, and with the aim of eliminating ovarian hyperstimulation syndrome. The good experience and high pregnancy rate in FET increased with greater use of pre-implantation genetic testing for aneuploidy. Several studies reported that FET achieved higher pregnancy rates and lower complication rates, as compared with fresh embryo transfers [18,19,20]. Various explanations were suggested, including non-physiologic estrogen levels in the fresh transfers, which caused abnormal placentation [21].

In naturally conceived pregnancies, the ‘‘window of implantation” is the time when the endometrium is most suitable to support trophoblast-endometrial interactions and invasion of the embryo. The window of implantation is thought to occur during a limited period around days 19–24 of an ideal 28-day cycle [22]. Substantial endogenous hormonal changes occur in the endometrium during the cycle, leading to endometrial receptivity and ending with embryo implantation. The major changes in the endometrial transformation towards decidualized endometrium include glycogen accumulation, cytokine, and growth factor secretions and the appearance of pinopodes. These secretory alterations during the luteal phase play an important role in receptivity, as they are thought to contribute to the active selection of embryos attempting implantation [23,24].

In ART, clinicians are challenged to imitate the naturally occurring steps in order to optimally prepare the endometrium to receive the transferred embryo. Studies on baboons demonstrated that high levels of estrogen have negative effects on the endometrium and on the ability of the trophoblast to invade uterine vessels during pregnancy [25,26,27]. Reflecting that observation on human endometrial cells [21] could explain the reports of higher rates of hypertensive disorders of pregnancy, low birth weights, and preterm deliveries in aFET cycles [28]. These findings indicate the importance of the treatment protocol used for FET.

### 4.1. Choosing the Treatment Protocol

The protocols we used for FET were tailored to address the specific cause of infertility and physiological alterations. Our FET cycle protocols are prescribed based on patients’ previous cycles, with the intention to start with the minimum amount of exogenous hormones. We endeavor to follow the physiological changes occurring in the endometrium before embryo implantation. A hormone replacement protocol is very common for FET and is the most acceptable regimen, especially for anovulatory patients. However, for these patients, ovulation induction may induce normal ovulation and enable FET cycles based on a more natural physiological response.

### 4.2. aFET vs. Ovu-FET

Several studies reported better cycle outcomes with Ovu-FET [29,30,31,32]. Our results support these later studies. In our practice, Ovu-FET resulted in significantly higher chemical and clinical pregnancy rates than aFET did. This can be explained in part by an unreceptive or out-of-phase endometrium affected by the exogenous hormones administered to patients in the aFET cycles. Previous data by Hromadová et al., support this assumption. They used an endometrial receptivity analysis to demonstrate that as many as 48.6% of women undergoing aFET demonstrated a displaced implantation window [33]. Previous studies suggested a benefit of using mild stimulation of induction, either with low-dose gonadotropins or aromatase inhibitors [34,35,36].

Melnick et al. compared pregnancy outcomes between natural cycle FET and aFET in patients undergoing euploid blastocyst transfers. They reported that implantation, clinical, and ongoing pregnancy rates were found to be significantly higher in ovulatory patients undergoing the natural cycle FET as compared to aFET for anovulatory patients [37]. In accordance with the previous study, our ongoing pregnancy rate and live birth rate were higher in Ovu-FETs and the miscarriage rate was significantly lower. These differences may be explained due to supraphysiologic estrogen levels that cause abnormal placentation and embryo invasion.

We found that despite a transfer of significantly lower quality embryos based on morphokinetic scoring in the Ovu-FET, we achieved significantly higher clinical pregnancy, ongoing pregnancy, and live birth rates. This accomplishment was consistent in both non-induced Ovu-FET and letrozole-induced Ovu-FET (Table 1). These results support the advantage of ovulatory cycles over aFET for patients with irregular cycles. We can deduce from these results that patients with lower quality ovulation, such as irregular ovulation or advanced maternal age, may benefit from letrozole-induced ovulation to achieve Ovu-FET. Induction of ovulation may overcome subtle defects in folliculogenesis, potentially correct the luteal phase, and hence, improve endometrial receptivity in selected patients.

Our study followed pregnancy outcomes, and no fetal anomalies were documented in our study group, supporting the safety of letrozole [38,39,40,41,42]. Our study patients did not demonstrate any significant differences in pregnancy complications in either the aFET or the letrozole group. This finding may be attributed to the small group sizes.

### 4.3. Letrozole-Induced Ovu-FET vs. Non-Induced Ovu-FET

We compared letrozole-induced ovulation to spontaneous ovulation. In this sub-cohort, we saw a significant difference in the distribution of the causes of infertility. Patients with irregular ovulation were part of the letrozole group only. Interestingly, even though we had a difference in distribution among patients, we achieved similar pregnancy rates, which indicate the advantage of letrozole for patients with irregular ovulation.

### 4.4. FET in PCOS Patients

Patients presenting with PCOS are usually prescribed a hormone replacement protocol. However, for these patients, induction of ovulation may induce normal ovulation and physiological FET, which may result in better pregnancy rates. Letrozole was shown to successfully and safely induce ovulation in fertility treatments and insemination [38]. Here, we report significantly better pregnancy rate in FET treatments for PCOS patients (Table 1).

Young women with regular menstrual cycles may benefit from natural cycles or modified natural cycles. Conversely, for advanced maternal age, natural cycles may harbor a disadvantage due to inferior corpus luteum function, which may cause dis-synchronization between the embryo’s developmental age and endometrial receptivity. To overcome this drawback, in natural cycles for advanced maternal age, the addition of ovulation triggering with hCG to support the corpus luteum may improve luteal support.

The optimal protocol for endometrial preparation for FET was evaluated in different studies and there is an ongoing debate whether frozen embryos transferred at ‘more physiologic hormonal levels’ which are not stimulated may result in higher pregnancy rates and fewer maternal and fetal complications compared to hormonal treatment protocols or artificial protocols that are not physiologic. Pregnancy complications such as hypertensive disorders were significantly lower in ovulatory cycles, probably due to the presence of corpus luteum and its secretions of relaxin and endothelial growth factor [43,44].

Conversely, studies by Kawamura et al. [45] and Kim et al. [46] indicated that the type of endometrial preparation does not have a statistically significant effect on the clinical outcomes of FET. In a prospective, randomized study that evaluated Ovu-FET vs. aFET, no superiority was defined for any specific protocol [47]. The review by Mackens et al. could not recommend any specific protocol for FET either [10]. A Cochrane review from 2017 evaluated randomized studies that could not reach a conclusion and summarized that the “optimal” treatment protocol has yet to be determined [7]. The current study, in contrast, demonstrated better outcomes in Ovu-FET compared to aFET in univariate analysis and in multivariate analysis.

Importantly, anovulatory PCOS patients achieved a significantly higher clinical pregnancy rate in the letrozole-induced Ovu-FET compared to aFET (44% vs. 22.5%, *p* = 0.044). Although the group is not large, this may suggest that letrozole can be considered a good treatment option for anovulatory PCOS patients.

A finding worth noting is the difference in endometrial thickness between the letrozole group and natural cycles. This may be explained by delayed elevation of peripheral estradiol in the letrozole group, which led to significantly thinner endometrium at the final US examination, prior to the decision on transfer timing [48]. However, this did not affect cycle outcomes.

Another interesting finding is the difference in neonatal birth weights, which were higher in the non-induced ovulation cycles compared to letrozole (3266 ± 446 g vs. 2617 ± 1106 g, *p* = 0.008). In both groups, the average birth weight was appropriate for gestational age and no maternal or fetal complications were observed, which decreased the clinical relevance of that finding. We hypothesize that the differences in neonatal weight may be explained due to the significant differences in maximal endometrial thickness (letrozole group 8.1 ± 1.9 vs. natural cycle 9.2 ± 2.2, *p* < 0.001; Table 3). However, we could not find any references to support this assumption. We believe that the difference between hormonal preparation and completely natural cycles for endometrial preparation may be the cause for sub-normal placentation, which may contribute to the difference in neonatal birth weights.

The disadvantages of our study are that it was retrospective. As a consequence, the Ovu-FET group included a heterogeneous mix of patients, which reduced the ability to draw substantial conclusions.

However, the strengths of this study are the large number of cycles. Outcomes were adjusted for potential confounders, including the treatment protocol for FET, maternal age, BMI, endometrial thickness, embryo quality at the transfer, and number of transferred embryos. The only parameters we found to significantly impact pregnancy rates were the treatment protocol, where ovulatory cycles were significantly better than artificial cycles (OR = 1.896, 95%CI = 1.31–2.73; *p* = 0.001) and letrozole treatment was significantly better compared to artificial cycles (OR = 2.029, 95%CI = 1.210–3.402; *p* = 0.007). Maternal age demonstrated OR = 0.967 (95%CI = 0.942–0.993; *p* = 0.015) and number of transferred embryos (OR = 1.518, 95%CI = 1.004–2.297; *p* = 0.048) for each additional embryo transferred. Moreover, our study strengthens the advantages of inducing ovulation in Ovu-FET compared to aFET and illuminates the benefits of using letrozole for PCOS patients. In addition, we report pregnancy outcomes and delivery rates, two topics in which the literature is deficient.

In conclusion, anovulatory PCOS patients may benefit from letrozole ovulation induced FET cycles, with results comparable to those of the general population, which demonstrated significantly better FET outcomes in ovulatory cycles compared with aFET. Generally, we found significantly higher clinical pregnancy rates in the ovulatory cycle FET, regardless of whether the mode of ovulation was spontaneous or induced. Additional randomized controlled trials are needed to establish the superiority of letrozole and Ovu-FET over aFET.

## Figures and Tables

**Table 1:** Patient characteristics and treatment outcomes according to the FET protocol.

Characteristics and Treatment Outcomes	aFET	Ovulatory Cycle-FET (*n* = 286)	*p*-Value
	(*n* = 348)		
Body mass index, kg/m^2^	26.1 ± 5.7	25.3 ± 5.4	0.088
Age, years	35.4 ± 6.5	35.3 ± 6.3	0.76
Infertility			0.66
Primary	96 (28%)	84 (29%)	
Secondary	251 (72%)	202 (71%)	
Parity			0.42
No	190 (55%)	147 (51%)	
Yes	157 (45%)	139 (49%)	
Smoker	56 (20%)	47 (19%)	0.91
Alcohol consumption	2 (1%)	5 (3%)	0.26
Cause of infertility (%)			
Unexplained	9.4	5.3	0.062
Male factor	45.9	53.8	0.15
Oligo-ovulation	24.2	10.6	<0.0001
Mechanical factor	19	26.5	0.062
Endometriosis	1.5	3.8	0.11
Number of embryos transferred, mean ± SD	1.21 ± 0.46	1.22 ± 0.45	0.22
Last estradiol before transfer	159 (59.7–283)	205 (127–306)	0.003
Last progesterone before transfer decision	0.21 (0.1–0.4)	0.78 (0.29–1.32)	<0.0001
ESHRE score of the transferred embryos, average	2.38 ± 0.86	2.21 ± 0.71	0.042
KID score of the transferred embryos, average	4.68 ± 0.64	4.63 ± 0.67	0.43
ESHRE score for all transferred embryos, average	2.37 ± 0.81	2.17 ± 0.71	0.031
KID score for all available embryos, average	4.67 ± 0.63	4.61 ± 0.64	0.44
Endometrial thickness before transfer (mm)	9.15 ± 2.2	8.84 ± 2.2	0.25
Positive beta hCG per transfer	119/348 (34.2%)	131/286 (45.8%)	0.003
Clinical pregnancy per transfer	95/348 (27.3%)	118/286 (41.3%)	<0.0001
Delivery and ongoing pregnancy	67/348 (19%)	94/286 (33%)	<0.0001
Ongoing pregnancy	31/348 (9%)	43/286 (15%)	0.02
Delivery rate	36/348 (10%)	51/286 (18%)	0.007
Abortion	52/95 (54.7%)	37/118 (33%)	<0.0001
Ectopic pregnancy	2/95 (2%)	4/118 (3%)	0.5
Neonatal birth weight (grams)	3364 ± 497	3130 ± 664	0.13
Fetal anomalies	0	0	NS
Hypertension	1	4	NS
Preterm delivery	1	2	NS
Diabetes	5	3	NS

**Table 2:** Patient characteristics and treatment outcomes: aFET vs. Letrozole-induced FET.

Characteristics and Treatment Outcomes	aFET	Letrozole-Induced FET	*p*-Value
	(*n* = 348)	(*n* = 80)	
Body mass index, kg/m^2^	26.1 ± 5.7	25.7 ± 6.1	0.62
Age, years	35.4 ± 6.5	35.5 ± 6.1	0.93
Infertility			0.58
Primary	96 (28%)	25 (31%)	
Secondary	251 (72%)	54 (69%)	
Parity			0.017
No	190 (55%)	56 (70%)	
Yes	157 (45%)	24 (30%)	
Smoker	56 (20%)	21 (31%)	0.018
Cause of infertility (%)			
Unexplained	9.4	3.8	0.24
Male factor	45.9	47.5	0.37
Oligo-ovulation	24.2	31.3	0.65
Mechanical factor	19	20	0.51
Endometriosis	1.5	2.5	0.35
Number of transferred embryos	1.21 ± 0.46	1.27 ± 0.51	0.31
ESHRE score of the first transferred embryo, average	2.38 ± 0.86	2.25 ± 0.67	0.13
KID score of the first transferred embryo, average	4.68 ± 0.64	4.57 ± 0.84	0.67
ESHRE for all available embryos, average	2.37 ± 0.81	2.20 ± 0.65	0.09
KID for all available embryos, average	4.67 ± 0.63	4.57 ± 0.84	0.67
Endometrial thickness, mm	9.15 ± 2.2	8.12 ± 1.9	0.003
Positive beta hCG per transfer	119/348 (34.2%)	36/80 (45.0%)	0.073
Clinical pregnancy per transfer	95/348 (27.3%)	33/80 (41.3%)	0.021
Ongoing pregnancy and delivery	67/348 (19%)	24/80 (30%)	0.04
Ongoing pregnancy	31/348 (9%)	13/80 (16%)	0.05
Delivery rate	36/348 (10%)	11/80 (14%)	0.43
Abortion	52/95 (54.7%)	12/33 (36.3%)	0.06
Ectopic	2/95 (2%)	2/33 (6.6%)	0.29
Neonatal birth weight, grams	3364 ± 497	2617 ± 1106	0.08

**Table 3:** Patient characteristics and treatment outcomes in NC-FET and letrozole-induced ovulation compared with non-induced ovulation.

Characteristics and Treatment Outcomes	Letrozole-Induced Ovulation	Non-Induced NC-FET	*p*-Value
	(*n* = 80)	(*n* = 196)	
Body mass index, kg/m^2^	25.7 ± 6.1	25.2 ± 5.1	0.42
Age, years	35.5 ± 6.1	35.0 ± 6.4	0.61
Infertility			0.88
Primary	25 (31%)	58 (29%)	
Secondary	54 (69%)	114 (71%)	
Parity			0.001
No	56 (70%)	90 (43%)	
Yes	24 (30%)	112 (57%)	
Smoker	21 (31%)	24 (14%)	0.001
Cause of infertility (%)			
Unexplained	3.8	5.8	1
Male factor	47.5	60.2	0.89
Oligo-ovulation	31.3	0	<0.0001
Mechanical factor	20	29.2	0.35
Endometriosis	2	4.8	0.73
Number of transferred embryos	1.27 ± 0.51	1.18 ± 0.39	0.27
ESHRE score of the transferred embryos, average	2.25 ± 0.67	2.20 ± 0.76	0.87
KID score of the transferred embryos, average	4.57 ± 0.84	4.68 ± 0.47	0.85
Total ESHRE for all available embryos, average	2.20 ± 0.65	2.19 ± 0.77	0.79
Total KID score for all available embryos, average	4.57 ± 0.84	4.63 ± 0.52	0.64
Endometrial thickness, mm	8.1 ± 1.9	9.2 ± 2.2	0.001
Positive beta hCG per transfer	36/80 (45.0%)	93/196 (47.4%)	0.79
Clinical pregnancy per transfer	33/80 (41.3%)	83/196 (42.3%)	0.89
Ongoing pregnancy and delivery	24/80 (30%)	68/196 (35%)	0.48
Ongoing pregnancy	13/80 (16%)	29/196 (15%)	0.85
Delivery rate	11/80 (14%)	39/196 (20%)	0.3
Abortion	7/33 (9%)	13/83 (7%)	0.47
Ectopic	2/33 (6%)	2/83 (2.5%)	0.33
Neonatal birth weight (grams)	2617 ± 1106	3266 ± 446	0.008

**Table 4:** Anovulatory PCOS characteristics and treatment outcomes between artificial (aFET) and letrozole-induced ovulation.

Characteristics and Treatment Outcomes	a-FET	Letrozole- Induced FET	*p*-Value
	(*n* = 80)	(*n* = 25)	
Body mass index, kg/m^2^	28.0 ± 5.6	30.0 ± 7.4	0.16
Age, years	33.4 ± 5.3	35.2 ± 5.3	0.14
Infertility			1
Primary	24 (30%)	7 (28%)	
Secondary	56 (70%)	18 (72%)	
Parity			0.49
No	47 (59%)	17 (68%)	
Yes	33 (41%)	8 (32%)	
Smoker	11 (16.4%)	5 (23.8%)	0.52
Number of embryos per transfer	1	1	--
ESHRE score of the transferred embryos, average	2.62 ± 1.06	2.40 ± 1.26	0.68
KID score of the transferred embryos, average	5.00 ± 0	4.50 ± 1.58	0.37
ESHRE score of all available embryos, average	2.50 ± 0.75	2.06 ± 0.69	0.2
KID score of all available embryos	4.75 ± 0.46	4.80 ± 0.63	0.49
Endometrial thickness (mm)	9.73 ± 2.96	8.18 ± 2.16	0.057
Positive beta hCG per transfer	30/80 (37.5%)	12/25 (48.0%)	0.36
Clinical pregnancy per transfer	18/80 (22.5%)	11/25 (44.0%)	0.044
Ongoing pregnancy and delivery	10/80 (12.5%)	7/25 (28%)	0.11
Ongoing pregnancy	6/80 (7.5%)	1/25 (4%)	1
Delivery rate	4/80 (5%)	6/25 (24%)	0.011
Abortion	7/18 (39%)	2/11 (18%)	0.24
Ectopic	1/18 (5%)	2/11 (18%)	0.27
Neonatal birth weight (grams)	3093 ± 561	2979 ± 618	0.39

## Data Availability

Data will be made available upon reasonable request to the corresponding author.
